# Computational Intelligence Techniques in Medicine

**DOI:** 10.1155/2015/196976

**Published:** 2015-03-05

**Authors:** Ezequiel López-Rubio, David A. Elizondo, Martin Grootveld, José M. Jerez, Rafael M. Luque-Baena

**Affiliations:** ^1^Department of Computer Languages and Computer Science, University of Málaga, 29071 Málaga, Spain; ^2^DIGITS, Faculty of Technology, De Montfort University, The Gateway, Leicester LE1 9BH, UK; ^3^Leicester School of Pharmacy, Faculty of Health and Life Sciences, De Montfort University, The Gateway, Leicester LE1 9BH, UK; ^4^Department of Computer and Network Systems Engineering, University of Extremadura, 06800 Mérida, Spain

## 1. Introduction

The advent of the information age, also commonly known as the digital age, has made a profound impact on health sciences. Vast amounts of datasets now flow through the different stages of healthcare organizations, and there is a major requirement to extract knowledge and employ it to improve these centres in all respects.

Intelligent computer systems provide support to health professionals involved both in the medical and managerial contexts. Amongst these systems, computational intelligence approaches have gained increasing popularity given their ability to cope with large amounts of clinical data and uncertain information.

The goal of this special issue is to offer a broad view of this exciting field, the ever-growing importance of which is driven by the increasing availability of data and computational power.

## 2. Computational Intelligence

Computational intelligence is based on biologically inspired computational algorithms. The key pillars that compose this field are neural networks, genetic algorithms, and fuzzy systems. Neural networks are algorithms that can be used for function approximation or classification problems [1, 2]. They include supervised, unsupervised, and reinforcement learning [3]. Genetic algorithms, however, [4] are search algorithms inspired by biological genetics. They rely on two main operators, cross-over and mutation. Populations of individuals representing solutions to the problem are created over several generations. The algorithm uses a random-guided approach to optimize problems based on a fitness function. Fuzzy logic [5] is based on fuzzy set theory [6] in order to encompass reasoning that is fluid or approximate rather than fixed and exact. Fuzzy logic variables have “truth” values ranging in degree between 0 and 1 which can also handle “partial truth”.

Computational intelligence techniques have been successfully used in many “real-world” applications in a variety of engineering problems. They can also be used in the medical research domain.

## 3. Organisation and Plan of the Special Issue

This special issue presents a compilation of research papers describing novel recent strategies and developments on the use of computational intelligence techniques in medicine. It will be of interest to researchers interested in industry, academics, and also to postgraduate students interested in the latest advances and developments in the field of computational intelligence in medicine. Its contents are summarized next.

One of the best-known application fields of these techniques is computer-aided diagnosis (CAD). At this point, two types of methods must be distinguished: those which aim at providing clinicians with enhanced diagnostic procedures that can lead to improved human decisions, and those that intend to provide a second opinion; that is, given a set of signs, they can indicate or confirm one or more possible disease processes.

Many methods belonging to the first type correspond to image processing algorithms. Indeed, the automated segmentation of magnetic resonance images (MRI) of the brain is employed to determine pathological regions and to plan image-guided surgery. On the other hand, high-resolution computed tomography has become a standard tool to diagnose diffuse lung diseases, and sparse representations can help to identify abnormal patterns. Segmentation is also useful at the microscopic scale, where intracellular calcium variation can be measured by a marked controlled watershed transform. Additionally, the emergence of a number of differing medical imaging techniques brings our attention to the problem of fusing these images. An approach based on the Nonsubsampled Contourlet Transform, which is a directional multiresolution image representation method, is also proposed.

Other diagnostic enhancement procedures are not related to image processing. In this issue, the most relevant criteria to diagnose Guillain-Barré syndrome are studied by the Partitions around Medoids clustering algorithm, which is able to manage both categorical and numerical data since it only requires the distance matrix amongst the samples. The reactivity of blood pressure (BP) to talking episodes is predicted by a hybrid system composed of several soft computing approaches, specifically neural networks, an adaptive neurofuzzy inference system, and support vector machines (SVMs). This increases the precision of BP measures for clinical and research purposes. Screening for prediabetes is carried out by means of a combination of neural networks and SVMs, which is aimed at early intervention to avoid the serious complications associated with the disease. Validation procedures for electroencephalographic (EEG) signal processing by principal component analysis (PCA) are also proposed, which is a valuable tool for many neurological studies. EEG signals are also analyzed by mixed-norm regularization for sensor selection in brain computer interfaces. Finally, the problem of privacy preservation in self-helped medical diagnosis is addressed, where secure two-party computation in wireless sensor networks is ensured in order to develop a system where the patient inserts a health card into an automated teller machine and obtains a diagnostic report.

The second class of methods includes classification systems which are most useful for the early diagnosis of diseases for which there is currently no definitive diagnostic test. Computer-aided screening for Alzheimer's disease based on neuropsychological rating scales is carried out by a hybrid approach which combines rough sets, genetic algorithms, and Bayesian networks, and an expert system for multicriteria decision support in psychotic disorders is proposed. Clinical decision support systems are not limited to diagnosis, as noted in the osteoporosis case, where the system can also recommend treatments and assess the fracture risk.

Management and planning represent the other sections of healthcare organizations that can also benefit from the applications of computational intelligence. Particle swarm optimization can be employed to enhance blood assignment in blood banks, whilst social network simulation can help to develop effective preventative and interventional strategies for AIDS epidemics.

Last but not least, computational intelligence techniques are also useful tools for probing the improvement of drug manufacturing processes, as demonstrated in another proposal where neural networks are applied to drug tablet visual tracking for high speed mass production.

*Ezequiel López-Rubio* *Ezequiel López-Rubio* *David A. Elizondo* *David A. Elizondo* *Martin Grootveld* *Martin Grootveld* *José M. Jerez* *José M. Jerez* *Rafael M. Luque-Baena* *Rafael M. Luque-Baena*

## References

[1] Gurney K. (1997). *An Introduction to Neural Networks*.

[2] Haykin S. (1999). *Neural Networks: A Comprehensive Foundation*.

[3] Masters T. (1993). *Practical Neural Network Recipes in C++*.

[4] Goldberg D. (1989). *Genetic Algorithms in Search, Optimization, and Machine Learning*.

[5] Zadeh L. A. (1965). Fuzzy sets. *Information and Computation*.

[6] Zadeh L. A. (1968). Fuzzy algorithms. *Information and Computation*.

